# Technologies for Medication Adherence Monitoring and Technology Assessment Criteria: Narrative Review

**DOI:** 10.2196/35157

**Published:** 2022-03-10

**Authors:** Madilyn Mason, Youmin Cho, Jessica Rayo, Yang Gong, Marcelline Harris, Yun Jiang

**Affiliations:** 1 School of Nursing University of Michigan Ann Arbor, MI United States; 2 School of Biomedical Informatics University of Texas Health Science Center at Houston Houston, TX United States

**Keywords:** medication adherence, technology assessment, remote sensing technology, telemedicine

## Abstract

**Background:**

Accurate measurement and monitoring of patient medication adherence is a global challenge because of the absence of *gold standard* methods for adherence measurement. Recent attention has been directed toward the adoption of technologies for medication adherence monitoring, as they provide the opportunity for continuous tracking of individual medication adherence behavior. However, current medication adherence monitoring technologies vary according to their technical features and data capture methods, leading to differences in their respective advantages and limitations. Overall, appropriate criteria to guide the assessment of medication adherence monitoring technologies for optimal adoption and use are lacking.

**Objective:**

This study aims to provide a narrative review of current medication adherence monitoring technologies and propose a set of technology assessment criteria to support technology development and adoption.

**Methods:**

A literature search was conducted on PubMed, Scopus, CINAHL, and ProQuest Technology Collection (2010-present) using the combination of keywords *medication adherence*, *measurement technology*, and *monitoring technology*. The selection focused on studies related to medication adherence monitoring technology and its development and use. The technological features, data capture methods, and potential advantages and limitations of the identified technology applications were extracted. Methods for using data for adherence monitoring were also identified. Common recurring elements were synthesized as potential technology assessment criteria.

**Results:**

Of the 3865 articles retrieved, 98 (2.54%) were included in the final review, which reported a variety of technology applications for monitoring medication adherence, including electronic pill bottles or boxes, ingestible sensors, electronic medication management systems, blister pack technology, patient self-report technology, video-based technology, and motion sensor technology. Technical features varied by technology type, with common expectations for using these technologies to accurately monitor medication adherence and increase adoption in patients’ daily lives owing to their unobtrusiveness and convenience of use. Most technologies were able to provide real-time monitoring of medication-taking behaviors but relied on proxy measures of medication adherence. Successful implementation of these technologies in clinical settings has rarely been reported. In all, 28 technology assessment criteria were identified and organized into the following five categories: *development information*, *technology features*, *adherence to data collection and management*, *feasibility and implementation*, *and acceptability and usability*.

**Conclusions:**

This narrative review summarizes the technical features, data capture methods, and various advantages and limitations of medication adherence monitoring technology reported in the literature and the proposed criteria for assessing medication adherence monitoring technologies. This collection of assessment criteria can be a useful tool to guide the development and selection of relevant technologies, facilitating the optimal adoption and effective use of technology to improve medication adherence outcomes. Future studies are needed to further validate the medication adherence monitoring technology assessment criteria and construct an appropriate technology assessment framework.

## Introduction

### Background

Accurately measuring and monitoring patient medication adherence is critical in clinical practice and research settings but continues to be a challenging task globally [1]. Various methods are used to measure medication adherence, such as patient self-reports, pill counts, pharmacy refill records, drug metabolites or biomarker testing, and directly observed therapy (DOT) [1]. However, none of these methods have been accepted as a standard measure of medication adherence across a variety of settings [2]. More recently, sensor technologies have been increasingly used to track the medication-taking behaviors of patients [1]. For example, the Medication Event Monitoring System (MEMS) can record every time the patient opens the pill bottle via a sensor embedded in the pill cap [3,4]. Such technologies provide a unique opportunity to measure and monitor patient medication adherence over time [1]. The notion that medication adherence monitoring technology represents the *gold standard* of measurement of patient medication adherence has been voiced by some researchers [3-9] but continues to be disregarded by others [10-15]. There is limited consensus on how to determine or select the appropriate medication adherence monitoring technology for use, which may be due to the lack of appropriate technology assessment criteria in this field.

The advantages and limitations of the commonly used methods for measuring medication adherence have been described in the literature. For example, DOT allows for direct observation of patient medication-taking actions [16-18], but it is expensive to sustain and produces a constrictive time strain on both health care providers (HCPs) and patients’ daily schedules [1,12,18,19]. As a common way to measure medication adherence, patient self-reporting respects patient autonomy but carries the potential risk of patient overestimation or underestimation of their adherence abilities [20-22]. Medication adherence monitoring technologies with various types and features are being continuously developed and upgraded [1]. Some newly developed technologies may possess unique features that are unfamiliar to users [23]. Despite this literature, there is no summary or synthesis that reflects a clear understanding of the characteristics and values of a variety of medication adherence monitoring technologies. There is a growing need for technology assessment criteria to guide the development and selection of appropriate technologies for monitoring medication adherence to improve patient outcomes [24].

Stakeholders’ expectations regarding the use of health information technology for monitoring medication adherence also vary. From a clinical practice perspective, a user-friendly interface and the accurate monitoring of adherence are considered when selecting appropriate monitoring technologies [1]. From the technological development perspective, although system accuracy and data fidelity remain high priorities, developers also need to consider the feasibility of technical engineering of the system, such as energy consumption and battery lifetime [25]. Advanced medication adherence monitoring technologies may not be limited to a single method to gather patient medication adherence information [1]. In addition, human interactions with these technologies can be complicated owing to the comprehensive medical and pharmacological contexts, as well as multidimensional patient medication adherence behaviors [22]. A compiled summary and assessment of currently available applications of medication adherence monitoring technologies is important for a better understanding of their capacities and performance when making decisions for their adoption and use.

### Objectives

The purpose of this narrative review is to summarize literature reports on the current applications of medication adherence monitoring technologies and identify potential assessment criteria to support decisions related to technology development and adoption.

## Methods

### Literature Search

PubMed, Scopus, CINAHL, and ProQuest Technology Collection databases were searched because of their broad collection of literature focusing on health, health care, and technological domains. A combination of search terms was included as follows: (*medication adherence*) AND (*measurement technology* OR *monitoring technology*). A full list of search strategies used for each database is included in Multimedia Appendix 1. To gather the most recent collection of medication adherence monitoring technologies, searches were focused on scholarly articles published between January 2010 and June 2021 and written in English.

### Eligibility Criteria

Studies were included in this narrative review if they met the following criteria: (1) described the development of medication adherence monitoring technologies, (2) assessed the characteristics of medication adherence monitoring technologies, or (3) tested the application of technologies for monitoring medication adherence. All study methods were included. Only articles published in English with their full text available were included. Considering the ease of dispensing medication and self-administration of pill form of medications, we focused on medication adherence technologies suited for pills. Medication adherence monitoring technologies that suited nonpill forms of medications, such as inhalers, eye drops, and injectable medications, were excluded. Studies that did not provide adequate descriptions of technology characteristics or used technologies that did not monitor patient medication adherence were also excluded. Study selection was performed manually using this set of eligibility criteria.

### Data Extraction and Information Synthesis

Data concerning medication adherence monitoring technologies were manually extracted from the reviewed articles by the first author (MM) and discussed with the research team. These elements included the following: (1) type of technology, (2) name of technology, (3) technical features, (4) data capture and applications, (5) perceived advantages, and (6) limitations of the identified technologies. Data directly related to publications, such as the country and publication year, were also gathered. Information regarding adherence monitoring technologies was extracted and organized into a table for further synthesis (Multimedia Appendix 2 [1-6,9-13,15-18,20,26-99]).

A descriptive analysis of the characteristics of the selected studies was conducted. Key characteristics, including the technical features, data capture methods, advantages, and limitations of each technology type, were assessed and summarized. Common and recurring elements were coded and categorized as potential assessment criteria. All identified potential criteria were discussed and evaluated among the team members until a consensus was reached. The final criteria were organized into categories and subcategories and presented as a matrix.

## Results

### Study Selection and Characteristics

**Figure 1:**
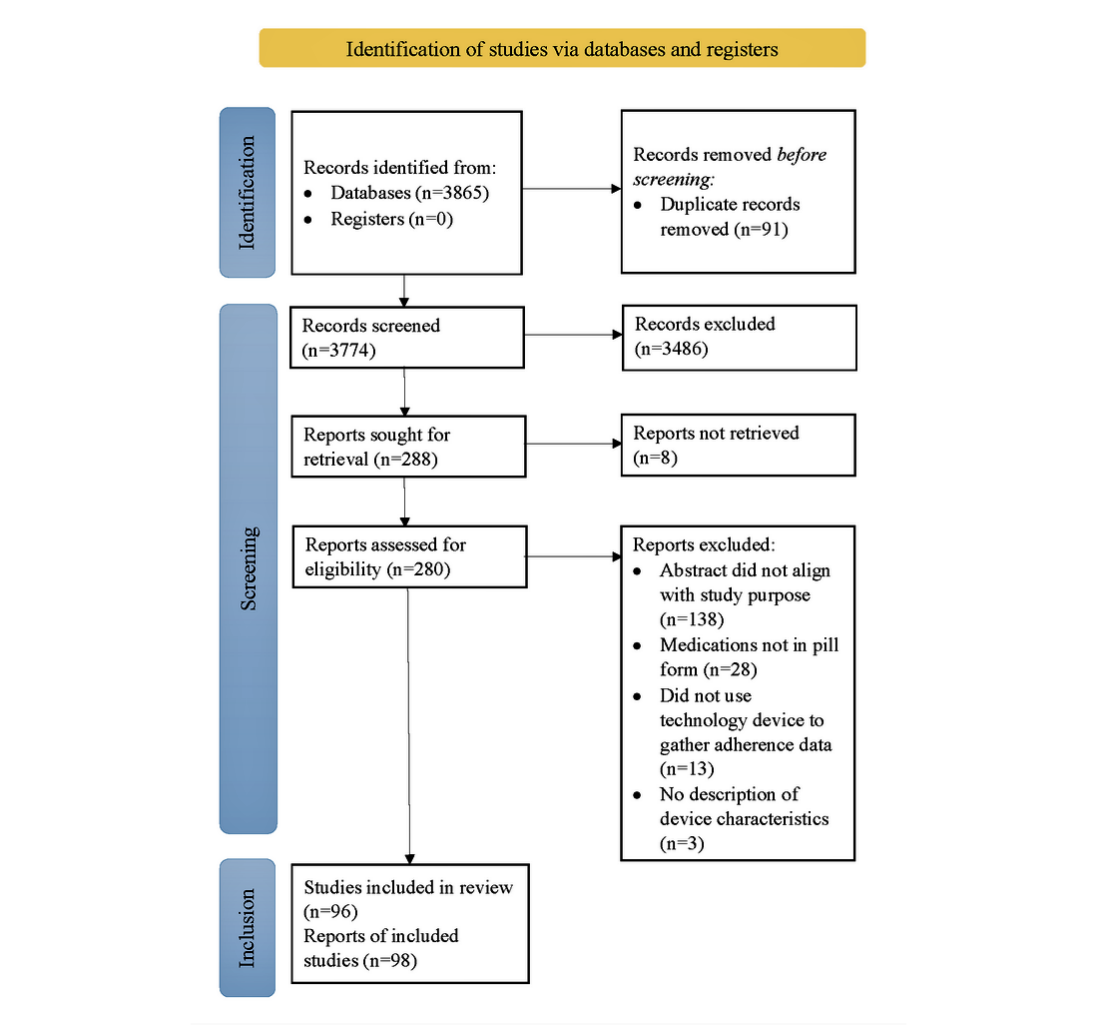
PRISMA (Preferred Reporting Items for Systematic Reviews and Meta-Analyses) diagram.

A total of 3865 records were retrieved from the database search. Of these 3865, the removal of duplicates left 3774 (97.65%) articles for title screening. After reviewing the titles and abstracts for relevance, 7.63% (288/3774) of the articles were identified for retrieval, of which 97.2% (280/288) were successfully gathered. Of the 288 articles, 8 (2.8%) articles were not retrievable because their full text was not available on the web. Following the assessment for eligibility via full-text review, 35% (98/280) of the articles were included in the final analysis. Figure 1 shows the PRISMA (Preferred Reporting Items for Systematic Reviews and Meta-Analyses) flowchart describing the overall search and selection process.

Among the reviewed articles, the vast majority (72/98, 73%) were published between January 2015 and June 2021. Over half (50/98, 51%) of the identified studies were published in the United States, followed by Canada (8/98, 8%), and Japan (4/98, 4%). The study types and designs varied greatly among the 98 reviewed articles. Most studies (41/98, 42%) were pilot tests of feasibility, acceptability, usability, or proof of concept. Only a few studies were randomized controlled trials, including pilot randomized controlled trials (5/98, 5%), retrospective cohort studies or secondary data analyses (6/98, 6%), or qualitative studies (8/98, 8%). Literature review articles (8/98, 8%), study protocols (4/98, 4%), and commentary and editorial comments (2/98, 2%) were included in the review analysis. The most common medications studied were tuberculosis treatment regimens (19/98, 19%) and antiretroviral therapy for HIV (16/98, 16%).

### Technology Types and Characteristics

#### Overview

A total of 81% (79/98) of applications of medication adherence monitoring technology were reported in the 98 reviewed articles. The identified technology types were categorized into eight major groups based on their technical designs and adherence monitoring functions*: electronic pillboxes or bags*, *electronic pill bottles*, *ingestible sensors*, *blister pack technology*, *electronic medication management systems*, *patient self-report–based technology*, *video-based technology*, and *motion sensor technology*. Table 1 shows the number of articles for each technology type group. As noted, some articles have reported more than one type of technology. The following sections outline the common defining technical features, data capture methods, and advantages and limitations gathered from the existing literature for each technology type.

**Table 1:** The number of publications by technology type (n=98).

Technology type	Publications, n (%)
Electronic pill boxes or bags	32 (33)
Electronic pill bottles	25 (26)
Ingestible sensors	22 (22)
Electronic medication management systems	12 (12)
Patient self-report technology	12 (12)
Blister pack technology	10 (10)
Video-based technology	7 (7)
Motion sensor technology	3 (3)

#### Electronic Pill Bottles

Consisting of a standard size pill bottle and an electronic cap that contains a microchip, this type of technology records a date-and-time stamp once the cap has been removed during an opening event. The transfer of adherence data depends on the version of the electronic pill bottle device. Some old versions of the MEMS often require manual download of the stored patient medication adherence data from the MEMS cap into the MEMS software through a small reader device [8-10,27,28]. Some newer versions of electronic pill bottle technologies, such as the GlowCap and AdhereTech devices, possess the ability to wirelessly transmit patient medication adherence data, providing opportunities to assess and monitor patient medication adherence in real time [1,15,18,29-31]. Electronic pill bottle technologies are commonly reported to have advantages including their discrete design and small size [10,16,32,33], objective medication adherence monitoring ability [1,8,11,14,34,35], and acceptance among patients [1,30,31]. However, as the pill bottle design is only capable of storing 1 type of medication at a time, these devices are not suitable for patients with complex multidrug regimens [1,4,8,9,18,30,32]. In addition, because the opening of the pill bottle is used as a proxy measure for adherence, patient actions such as failing to ingest removed medications, *pocket dosing* (taking out multiple doses of medications at one time), and *curiosity openings* (opening the device but not removing medications) can lead to inaccurate estimates of patient medication adherence [1,2,4,5,9-11,14-16,27,28,30,33,36,37].

#### Electronic Pill Boxes or Bags

Similar to the electronic pill bottle technology, electronic pill boxes or bags record a date-and-time stamp whenever they are opened. However, unlike electronic pill bottles, these technologies can often store multiple types or strengths of medication in various compartments within the device. The size and storage capacity vary among the different types of available electronic pill boxes or bags. Most of the identified electronic pill boxes or bags possessed the ability to transmit patient medication adherence data in real time via existing cellular networks [1,9,26-28,38-43,100], wireless Bluetooth [1,44,45], or general packet radio service [27,46,101,102]. One device required manual uploading of patient adherence data during clinic visits [47]. Although the capability of these devices to store multiple medications makes them better suited for complex multidrug regimens, this advantage is dependent on the device, as they can vary drastically in size and pill storage capacity. This was evident when examining the Wisepill device’s storage capacity of 60 small-sized pills [1,27,39,41,46] compared with MedTracker’s storage capacity of a week’s worth of medication [44]. However, larger-sized devices are often described as obtrusive [10,40,48] and have increased risks to patient privacy [1,39,40,49], thus limiting the acceptability of the device for patient populations, particularly for those who do not wish to disclose their health status (eg, HIV positive) to others [39]. Furthermore, these devices cannot directly confirm ingestion of medications, raising concerns toward their medication adherence monitoring accuracy because potential patient behaviors, including *pocket dosing* and *curiosity events*, may impede medication adherence rate estimates [1,10,15,16,26,27,30,38-40,44,48-50,101]

#### Blister Pack Technologies

All but 3 blister pack technology applications identified included an attachable adhesive label that contained a microchip and conductive wire pattern [4,36,51]. Removing medication from the blister pack created a break in the label circuit and was recorded by the microchip with a date-and-time stamp. Patient medication adherence data are wirelessly transmitted to central servers and are often accessible to HCPs, allowing for real-time adherence monitoring [1,6,51-55]. As the design of blister packs stores the appropriate dose of medication in singular pockets, each removed dose is registered as an individual event, thereby eliminating the potential for patient *pocket dosing* and *curiosity openings* [56]. However, currently within these devices, the action of removing a dose has been found to break the conductive tracks of the surrounding doses occasionally and accidentally, leading to the registration of multiple removal events, which decreases the accuracy of monitoring with these technologies [56,57]. Moreover, this method of medication adherence monitoring is a proxy measure and cannot confirm patient ingestion of medication, further limiting the accuracy of patient medication adherence estimates [1,4,52,55].

#### Ingestible Sensors

Ingestible sensors, otherwise known as digital pills [12] or digital ingestion monitoring [50], consist of a technological system that includes microsensors, an adhesive external monitor worn on the abdomen, and a mobile app. The microingestible sensors are coencapsulated with medication and ingested into the body, where stomach gastric fluids dissolve the capsule containing the medication and sensor. Activation of the sensor upon contact with gastric fluid transmits a unique signal to the external monitor. The detected ingestion event is transferred to a mobile app that uploads the event’s date-and-time stamp, along with other recorded physiological measures (eg, heartbeat), to a central server. These technological systems possess the advantage of direct observation of medication ingestion [3,13,15,16,30,37,58-65], as well as real-time adherence monitoring [1,3,9,12,30,37,49,58,61-66,103]. By directly identifying individual ingestion events of medication, these technologies can detect multiple ingestion events at a given time, thereby improving the accuracy of measuring patient medication adherence rates [60,61,65,103]. In addition, the ingestion event detection accuracy of ingestible sensors is high, with rates of 95% to 99.1% observed experimentally [3,58,65,103]. However, owing to the direct ingestion of technological sensors, concerns over patient privacy and autonomy are prominent because of the invasive nature of these devices [9,13,20]. Patient reports of skin irritation caused by the external monitor [1,12,40,59,61,63,64] and the possibility of sensor retention within the body [15,60,64] are considerable limitations of these technologies, as well as potential risks to patient health and safety.

#### Electronic Medication Management Systems

The devices identified within the category of Electronic Medication Management System (EMMS) vary in their functionalities, with reported advantages and limitations; however, all systems possess similar features that focus on aiding patients in their medication management and documenting their medication adherence patterns. Three novel EMMS devices that presented interesting functionality characteristics included the radio frequency identification (RFID)-based medication adherence intelligence system [44,67], ReX (DosentRx Ltd) [68], and the Medication Behavior Monitoring System (MBMS) [69]. The RFID-based medication adherence intelligence system (RMAIS) is composed of an RFID reader, scale, microcontroller, liquid crystal display panel, and a motorized rotation platform [44,67]. The patient’s pill bottles are labeled with an RFID tag that stores the medication’s information, such as the medication name and appropriate dose [67]. At a scheduled medication administration time, the RMAIS generates audio medication reminders and rotates the correct pill bottle in front of the patient [44,67]. The scale underneath the rotation platform weighs the pill bottle, and the medication information is displayed using an RFID reader [44,67]. After the patient has removed the medication from the pill bottle, the scale measures the weight of the bottle and uses the difference in weight to determine the number of doses removed [44,67]. If the system detects events of nonadherence, an HCP is alerted [44,67]. An advantage of this system is that it provides guidance to patients who must navigate complex multidrug regimens by eliminating the need for patient decision-making concerning what medication to take, how much, and at what time [67]. However, because this system is also a proxy monitor of medication adherence and cannot confirm the actual ingestion of medication, its accuracy is consequently limited [44].

ReX is a recently developed device composed of a reusable drug dispensing unit, disposable cassette, mobile app, and a Dose-E Analytics cloud system [68]. The patient’s medication is stored inside the device and can only be released at the appropriate time, at the correct dose, and directly into the patient’s mouth [68]. The mobile app transfers patient medication adherence data from the drug dispensing unit to the Dose-E Analytics cloud system, which is accessible to HCPs, allowing real-time medication adherence monitoring [68]. A critical advantage of the device is the dispenser mechanism that prevents patient medication overadherence and administration of medication at incorrect time intervals [68]. However, even though the device can monitor the medication up until delivery into the patient’s mouth, it cannot confirm the actual ingestion of the medication, thereby inhibiting the accuracy of its medication adherence estimates.

Finally, MBMS devices use newly emerging technologies such as the Internet of Things, deep learning, and artificial intelligence [67]. The MBMS is unique in that it combines the following three categories of medication adherence monitoring technologies: electronic pillboxes, motion sensor technology, and video-based monitoring technology [67]. The device uses a set alarm to remind patients to take their medication [67]. As the patient approaches the device, motion sensors placed around the patient’s home detect the movement and signal the MBMS device to begin recording a video of the patient’s medication behavior [67]. Once the device recognizes the patient’s act of raising an arm to drink water, the internal pillbox that stores the medication releases the appropriate medication and quantity onto a platform with a scale [67]. The MBMS determines whether the patient takes the dispensed medication based on whether the scale converges to zero [67]. HCPs receive weekly adherence reports from the MBMS. Roh et al [69] found that when an MBMS device was used, medication adherence was higher than in patients who did not use the device. However, similar to RMAIS and ReX, the inability of the system to detect actual medication ingestion inhibits its potential accuracy in monitoring patient medication adherence.

#### Video-Based Monitoring Technology

Similar to DOT, where patients administer their medication in the presence of an HCP, most video-based adherence monitoring technologies use video cameras for patients to self-record medication ingestion event videos for retrospective analysis by HCPs or, in 2 unique cases, by artificial intelligence [70-72]. Video-DOT (VDOT) was the most common technological method for this category of technology. Patients either ingest their medication during a synchronous video call with their HCP or upload an asynchronous video for the HCP to review [16,18,48,73,74]. Real-time medication adherence monitoring is facilitated by the direct and continuous use of medication ingestion event observation by HCPs [49,74]. An additional advantage of VDOT is that, compared with DOT, VDOT is considered more flexible, cheaper, and less intrusive to HCPs and patients [16,49,73]. However, several potential limitations to VDOT include technical difficulties, such as poor video quality [73], trouble uploading ingestion event videos [16], and complications with video camera devices [74]. There is also a potential risk of patients forgetting to self-record as they ingest their medication, which may lead to inaccurate reports of medication adherence [16].

#### Motion Sensor Technology

Currently, the medication adherence monitoring motion sensor technologies that we have identified are still under development. Three individual adherence-monitoring motion sensor devices were found, yet all their functionalities were similar. These devices were worn on wrists and resembled the size of a wristwatch [13,75,76]. The wrist-worn devices were triaxial accelerometers that identified the medication administration movements of patients [13,75,76]. Patient medication adherence data were then stored and uploaded to an HCP-accessible database in real time [75]. Wang et al [13] reported a correct ingestion event detection rate of 84.17%. Given that the action of administering medication closely resembles other everyday actions such as eating, drinking, or wiping one’s mouth, the accuracy of these technological systems is currently limited [75]. Despite these limitations, motion sensor technologies possess the advantages of being noninvasive [75] and nonintrusive [76] methods of medication adherence monitoring.

#### Patient Self-report Technology

Similar to EMMS, patient self-report technologies vary in their specific functionalities, yet they all gather subjective medication adherence data by interacting with the patient via phone calls [16,18,26,38,49,53,77-80], smart buttons [55], eDiaries [81], web-based platforms [82,83], and mobile apps [84]. Patient adherence is available in real time for most self-reported devices [18,49,53,78-80,82-84]. Compared with objective adherence monitoring technologies, patient self-report technologies are lower in cost [26,53] and less stigmatizing [16]. Nevertheless, because this technological method of adherence monitoring is subjective, there is a high potential for inaccurate medication adherence reporting by patients, negatively impacting the accuracy of these technologies [16,38,78,79].

A summary of the defining characteristics, data capture methods, and use of data in patient adherence monitoring for each technology type is presented in Table 2. The full details are included in Multimedia Appendix 2.

**Table 2:** Summary of the defining characteristics, data capture methods, and use of data for patient medication adherence monitoring for each technology category.

Technology category	Defining characteristics	Data capture methods	Use of data for adherence monitoring
Electronic pill bottles	Standard size pill bottles with electronic caps that contain microchips to detect opening events	Opening events of the pill bottle are date-and-time stamped	Recorded opening events act as a proxy measure for medication ingestion
Electronic pill boxes and bags	Devices shaped as pill boxes or bags. Sizes of devices vary. Within each device is a microchip that detects opening events	Opening events of the device are date-and-time stamped	Recorded opening events act as a proxy measure for medication ingestion
Blister pack technologies	Most of these devices are attachable adhesive labels containing a microchip and conductive wire pattern applied to standard blister packs^a^	Breakages in the conductive wire track are recorded as *opening* events and date-and-time stamped	Recorded opening events act as a proxy measure for medication ingestion
Ingestible sensors	Pills embedded with ingestible microsensors that are paired with an external wearable sensor and mobile app	Contact with gastric environment activates microsensor which transmits a signal to the external monitor and is recorded with a date-and-time stamp	Direct measure of medication ingestion events
EMMS^b^	Devices that aim to aid patients in managing medication administration by controlling the type of medication, dosage, or timeframe that medications are accessible^a^	Systems dispense medications and record date-and-time stamps of these events. For example, using scales to detect differences in the device’s weight and calculating the amount of medication removed by the patient^a^	Most systems used technologies such as scales and medication dispensing events as proxy measures for medication ingestion
Video-based monitoring technology	Systems that used video cameras to capture patients’ medication ingestion events	Video recording of medication-taking events which are later verified by reviewers	Substitute for DOT^c^
Motion sensor technology	Devices are worn on the wrists and contain motion sensing gyrometers and accelerometers to detect patient medication-taking behaviors	Wearable gyrometers and accelerometers identify and record patient motions that match previously programmed medication-taking movements	Physical motions of patients used as a proxy for medication ingestion
Patient self-report technology	Devices that gather adherence data via patient reporting^a^	Patients report medication-taking events via phone calls or other electronic means, such as mobile apps or web-based platforms^a^	Patient reports act as subjective indicators of medication ingestion events

^a^More examples and the full list of features and functions is provided in Multimedia Appendix 2.

^b^EMMS: Electronic Medication Management System.

^c^DOT: directly observed therapy.

### Medication Adherence Monitoring Technology Assessment Criteria

#### Categories Identified

During the data extraction process, common characteristics, recurring elements, and the reported advantages and limitations of all medication adherence monitoring technologies were synthesized and categorized into a set of adherence monitoring technology assessment criteria. These assessment criteria were not categorized by technology type, as various potential assessment criteria were commonly expressed across technologies, suggesting the plausibility of general assessment criteria for all medication adherence monitoring technologies. All 28 specific criteria were included under the following five assessment categories: *development information*, *technology features*, *adherence data collection and management*, *feasibility and implementation*, *and acceptability and usability*. Each category possesses the main feature of interest that allows and supports medication adherence monitoring or measurement. A brief description of each assessment category is provided in the following sections.

#### Development Information

The development information category contains components related to the general development information of the medication adherence monitoring technology of interest. This category should include information regarding the developer, development stage, commercial availability, and regulatory approval status of organizations such as the Food and Drug Administration.

#### Technology Features

They contain criteria directly related to the technological setup of medication adherence monitoring technologies. This category includes the following two subcategories: device or hardware and system or software features. The assessment elements of device size, battery life, medication storage capacity, installation or software needs, and the need for wireless connection are considered device or hardware features. System or software feature assessment includes reminder and alert functions, device accommodation for complex medication regimens, and information technology support availability.

#### Adherence Data Collection and Management

This category pertains to methods for the capture of medication adherence data and the use of such data. This assessment category was subdivided into data collection and management categories. In data collection, the assessment focuses on subjective versus objective data collection, proxy data collection, date-and-time stamps, and the potential for data entry errors. Data management pertains to the assessment of transmission and upload methods, data display and summary, real-time monitoring capabilities, data accessibility by HCPs, and data security.

#### Feasibility and Implementation

This category focuses on the components necessary or related to the use of the technology in real-world settings. In addition to device cost efficiency, the interoperability of the technology with current clinical systems should also be considered.

#### Acceptability and Usability

This is the last category, examining the interaction and relationship between the technology of interest and technology users. These elements include ease of learning and use, device portability, potential risks to patient privacy, and technology-related harms, such as risks to patient health or safety.

All assessment categories and criteria are listed within an organized matrix structured to support technology development and adoption (Textbox 1).

**Medication adherence monitoring technology assessment criteria.**

**Development information**

- Developer
- Development stage
- Regulatory approval status
- Commercial availability

**Technology features**

- Device or hardware
  Size
  Battery life
  Storage capacity
  Installation or additional software needed
  Wireless connection needed
- System or software
  Reminder and alert function
  Accommodation for complex medication regimens
  Information technology support availability

**Adherence data collection and management**

- Data collection
  Subjective vs objective data collection
  Proxy data collection
  Date-and-time stamps
  Data entry error (eg, curiosity opening and sensor retention)
- Data management
  Data transmission and upload methods
  Data display and summary
  Real-time monitoring
  Data accessibility by health care providers
  Data security

**Feasibility and implementation**

- Cost efficiency
- Interoperability with current clinical systems

**Acceptability and usability**

- Ease of learning
- Ease of use
- Portability
- Risks to patient privacy
- Risks to patient health or safety (eg, skin rashes)

## Discussion

### Principal Findings

As the adoption and development of medication adherence monitoring technologies continue to increase, understanding their key characteristics is vital. This narrative review provides an overview of the technical features, data capture methods, and advantages and limitations of current medication adherence monitoring technologies reported in the literature and synthesizes 28 technology assessment criteria that can be used to guide the development and selection of relevant technologies. Overall, there were 8 types of medication adherence monitoring technologies, dominated by electronic pill bottles, electronic pill boxes or bags, and ingestible sensors. Although technical features varied by technology type, there were common expectations regarding the advantages of using these technologies for accurately monitoring medication adherence and increasing the adoption of these technologies in patients’ daily lives.

### Current Technology Characteristics

All current medication adherence monitoring technologies have varying degrees of technological restriction. The most commonly reported technology types, electronic pill boxes or bags and electronic pill bottles that use opening events as a proxy for medication ingestion, face undesired patient behaviors such as *pocket dosing* and *curiosity openings*, which are obstacles to the device’s accuracy for patient adherence estimates [2,9,11,14,16,27,30,33,37,41,48-50]. Despite this limitation, the popularity of developing and using pill monitoring devices remains, which may be due to their unobtrusiveness and convenience of use in patients’ everyday routines, suggesting an increasing adoption of objective measurement and monitoring of medication adherence through technological approaches.

Although electronic pill bottles possess extensive histories of being used in both clinical and research settings, the presence of many other medication adherence monitoring technology studies in the pilot and feasibility phase implies that the integration of newer technologies, such as motion sensor–based technologies and ingestible sensors, is still relatively new and ongoing [1,4,15,18,60,64,76]. Overall, technologies capable of monitoring patient medication adherence provide significant advantages, including real-time medication adherence data reporting, yet questions concerning the accuracy of these devices prohibit them from becoming a *gold standard* in clinical and research standings. Thus, until further developments in medication adherence monitoring technologies occur, multiple methods for patient medication adherence assessment must be used to evaluate patient medication adherence rates and behaviors [1].

Many medication adherence monitoring technologies possess software to organize patient medication adherence data to an extent; however, most of these devices require separate analysis and quantification of the data by HCPs or researchers [1], creating a significant burden of time consumption and the concern of further data integration with other technology applications. The development of a patient medication adherence data management software that can construct automatic visualizations of patient medication adherence estimates should be considered to provide an easy interpretation of patient medication adherence patterns. Moreover, the use of advanced software for adherence data processing and presentation may improve the adoption and integration of medication adherence monitoring technologies in clinical settings.

### Medication Adherence Data Capture and Use

In addition to variances in technical features, current medication monitoring technologies differ in their data capture methods and the subsequent use of such data in relation to patients’ medication adherence assessment. The ability of most medication adherence monitoring technologies to provide real-time observations of patient medication adherence behaviors is beneficial to HCPs and researchers to prevent nonadherence and facilitate appropriate interventions [1,15,36,37,79]. However, most of these technologies rely on proxy measures of medication adherence, such as device opening events, thereby limiting their data accuracy [2,9,11,14,16,27,30,33,37,41,48-50]. Furthermore, successful implementation of these technologies in clinical settings or the integration of patient medication adherence monitoring data into clinical practice has rarely been reported. One of the major barriers is the interoperability of these monitoring technology systems with established clinical information systems and workflow. To facilitate the adoption of medication adherence monitoring technologies in clinical systems to improve patient care, the method of adherence data capture must be feasible for targeted patients and the acquired data must be easily integrated into standard electronic health record systems. The medication adherence data capture methods and data use presented in this review can help guide HCPs and researchers toward the appropriate selection of medication adherence monitoring technology. Developers must also consider the implications of medication adherence data capture within clinical and research settings to ensure greater ease of use for both patients and providers.

### Technology Assessment Criteria

To the best of our knowledge, this is the first collection of assessment criteria focused on technologies to monitor patient medication adherence. The proposed assessment criteria include five major categories as follows: development information, technology features, adherence to data collection and management, feasibility and implementation, and acceptability and usability. The identified criteria highlight significant aspects of medication adherence monitoring technologies that must be considered during technology development and adoption. For example, an important component of medication adherence monitoring technology implementation is cost; however, a common limitation of these technologies is their expensive price tags [1,2,5,7,9,12,18,27,38,54,78,85,102]. The proposed criteria emphasize the cost efficiency of medication adherence monitoring technologies within the feasibility and implementation category. The high cost of devices restricts their adoption in clinical and research settings because other methods, such as patient self-reports, are significantly cheaper [1,78].

As this compilation of assessment criteria was formed by reviewing the current literature, other existing challenges within technology acceptance or technology design features were also addressed, such as risks to patient privacy or the effect of large device size on user adoption owing to daily life inconveniences [25]. Given the multifaceted nature of the proposed assessment criteria, they can be used to guide the improvement of these technologies for better medication adherence measures and monitoring.

In addition, our set of proposed assessment criteria possessed a structure similar to that of other validated mobile health assessment frameworks. For example, a pyramid for app evaluation framework, proposed by Henson et al [104] and adapted by the American Psychiatric Association as the App Evaluation Model, introducing a similar 5-level structure of evaluation categories, including access and background, privacy and security, clinical foundation, usability, and data integration toward therapeutic goals [104,105]. Similarly, each category covers a few specific evaluation criteria; for example, ease of use is assessed under the usability category [105]. Certain general technology assessment criteria can be applied to both mobile health apps and medication adherence monitoring technology, such as usability, privacy and security, and data integration. However, adherence monitoring technology possesses technical features to support medication storage and management, which results in its unique assessment criteria, such as the medication storage capacity of the device or date-and-time stamps indicating medication-taking actions. The collection of medication adherence monitoring technology assessment criteria was generated from an extensive literature review and information synthesis, which demonstrates its solid evidence foundation but also suggests that further empirical tests and validation are needed in the future.

### Limitations

This narrative review has some limitations. First, our database selection and search strategies might not have been sufficiently extensive to capture all published literature. Moreover, we limited the studies to those published in English, potentially excluding other existing medication adherence monitoring technologies from non-English sources. The proposed medication adherence monitoring technology assessment criteria are representative of the elements identified in our literature review and synthesis, which are subject to further validation and evaluation. We did not review detailed information published by specific manufacturers. Finally, given that the scope of this review was focused on medication adherence technologies used for the monitoring of pill form medications, the assessment criteria and the rest of our findings may not be generalizable to all types of medication. It is noteworthy that a large proportion of the identified articles were pilot or feasibility studies. Consequently, our assessment domain of the criteria may also be limited to the early stages of technology development.

### Conclusions

Overall, this narrative review presents a summary of the current technological features and data capture methods, reports the advantages and limitations of medication adherence monitoring technologies for pill form medications, and proposes a potential technology assessment criteria. Our constructed assessment criteria are crucial for the development and adoption of these technologies. Specifically, further technological development is required to expand the interoperability of medication adherence monitoring technology systems in clinical settings. The increased implementation of technologies that monitor patient medication adherence has demonstrated the potential to improve patient medication adherence behaviors. Although this technological method of patient medication adherence monitoring cannot be defined as the *gold standard* method for medication adherence monitoring, the functionalities that they possess may improve patient medication adherence and support greater patient health outcomes over time.

## Appendix

Multimedia Appendix 1 Search strategies.

Multimedia Appendix 2 Summary of medication adherence monitoring technologies.

## Abbreviations

DOT: directly observed therapy
EMMS: Electronic Medication Management System
HCP: health care provider
MBMS: Medication Behavior Monitoring System
MEMS: Medication Event Monitoring System
PRISMA: Preferred Reporting Items for Systematic Reviews and Meta-Analyses
RFID: radio frequency identification
RMAIS: radio frequency identification–based medication adherence intelligence system
VDOT: video-directly observed therapy
