# Supplementary material for: Technologies for Medication Adherence Monitoring and Technology Assessment Criteria: Narrative Review
Source: JMIR Mhealth Uhealth. 2022 Mar 10;10(3):e35157. doi: 10.2196/35157 (PMC8949687; doi:10.2196/35157)
Supplement: Multimedia Appendix 1 [file mhealth_v10i3e35157_app1.doc]

Multimedia Appendix 1

Table 1. Search strategies.

|  | “Medication adherence” | “Monitoring technology” | “Measuring technology” |
| --- | --- | --- | --- |
| PubMed | “medication adherence”[MeSH] OR “medication adherence” OR  “medication compliance” OR “medication adherence”[tiab] “medication compliance”[tiab] | “monitoring technology” OR “monitoring device” OR “monitoring technology”[tiab] OR “monitoring device”[tiab] | “measuring technology” OR “measuring device” OR “measuring technology”[tiab] OR “measuring device”[tiab] |
| Scopus | TITLE-ABS-KEY (“medication adherence” OR “medication compliance”) | TITLE-ABS-KEY (“monitoring technology” OR “monitoring device”) | TITLE-ABS-KEY(“measuring technology” OR “measuring device”) |
| CINAHL | (MH “medication adherence”) OR (MH “medication compliance”) OR TI(“medication adherence” OR “medication compliance”) OR AB(“medication adherence” OR “medication compliance”) | (MH “monitoring technology”) OR (MH “monitoring device”) OR TI(“monitoring technology” OR “monitoring device”) OR AB(“monitoring technology” OR “monitoring device” | (MH “measuring technology”) OR (MH “measuring device”) OR TI(“measuring technology” OR “measuring device”) OR AB(“measuring technology” OR “measuring device”) |
| ProQuest Technology Collection | MESH(“medication adherence”) OR “medication compliance” OR AB,TI(“medication adherence OR “medication compliance”) | AB,TI(“monitoring technology” OR “monitoring device”) OR “monitoring technology” OR “monitoring device” | AB,TI(“measuring technology” OR “measuring device”) OR “measuring technology” OR “measuring device” |
