# Supplementary material for: Technologies for Medication Adherence Monitoring and Technology Assessment Criteria: Narrative Review
Source: JMIR Mhealth Uhealth. 2022 Mar 10;10(3):e35157. doi: 10.2196/35157 (PMC8949687; doi:10.2196/35157)
Supplement: Multimedia Appendix 2 [file mhealth_v10i3e35157_app2.docx]

Appendix Table 1. Summary of Medication Adherence Monitoring Technologies.

| ***Medication Adherence Monitoring Technologies*** | | | | | |
| --- | --- | --- | --- | --- | --- |
| **Device** | **Technical Features** | **Advantages** | | **Limitations** | **References** |
| ***Electronic Pill Bottles*** | | | | | |
| Medication Event Monitoring System (MEMS) | Consists of a pill bottle with an electronic microchip located in the cap. Records a date-and-time stamp each time the device is opened. Patient adherence data is downloadable onto a computer via USB. Must use MEMS software to access adherence data. | - Objective monitoring of patient adherence - Provides patterns of patient adherence - Wireless technology - Easy to use - Well accepted by patients - Respects patient autonomy - Long battery life - Feasibility established - May improve patient adherence behavior | | - Bulky size - Expensive - Inhibited accuracy due to “pocket dosing” and “curiosity openings” - Not compatible with medication meant for blister packaging - Not suitable for complex medication regimens - Cannot confirm medication ingestion - Device must be brought into clinic to manually upload adherence data - Requires training for data interpretation and software use - Time burden to synthesize adherence data results and patterns - Potential for Hawthorne Effect - Potential for technical malfunctions | [1,4,5,8-11,14-18,27,28,  35-37,75,  80] |
| Microelectronic Monitoring System (MEMS) | Electronic cap and normal sized medication pill bottle. Automatically records opening events with date-and-time stamps. | - Adherence data is precise and easily quantified - More accurate and valuable data compared to self-report - Provides patient adherence patterns and information about dosing schedule - Less subject to patient manipulation than self-report - May improve patient adherence behavior | | - Inhibited accuracy due to “pocket dosing” and “curiosity openings” - Expensive - Potential for Hawthorne Effect - Patient subject to take wrong dose of medication | [2] |
| Wireless Medication Event Monitoring System (MEMS) | Pill bottle with an electronic cap that contains a plunger mechanism. During an opening event, the plunger mechanism moves, and microcircuits record the date-and-time of the event. Patient adherence data is transmitted to a central database via wireless circuits. | - Real-time adherence monitoring - Patient adherence data accessible to HCPs/researchers in real-time - Long battery life of >24 months - Can store 3800 opening events - Fits to standard medication vials | | - Inhibited accuracy due to “pocket dosing” and “curiosity openings” - Expensive | [33] |
| Medication Event Monitoring System (MEMS6®) TrackCaps | Pill bottle device that uses a micro-electronic circuit to register and store date-and-time stamps of opening events | - Objectively monitors patient adherence - Long battery life of 36 months - Can store 3518 opening events | | - Device must be brought into clinic to manually upload adherence data - Potential technological malfunctions (reported rates of error: 0-24%) | [34] |
| GlowCap | Electronic medication pill bottle and cap that contains a microchip that detects opening events and records a date-and-time stamp for each event. Patient adherence data is wirelessly transmitted to a central server. Wirelessly sends medication alerts via mobile network to patients and HCPs/researchers. Generates a monthly adherence report that is mailed to HCPs/researchers. | - Real-time adherence monitoring - Patient adherence data accessible to HCPs/researchers in real-time - Well accepted by patients - Provides patient adherence patterns - Wireless technology - Non-stigmatizing compared to other medication adherence monitoring technologies - Provides medication adherence reminders | | - Inhibited accuracy due to “pocket dosing” and “curiosity openings” - Cannot confirm medication ingestion - Not compatible with complex medication regimens - Expensive - Time burden to synthesize adherence data results and patterns - Potential technical malfunctions - Patient privacy concerns - Limited medication storage capacity | [1,16,29, 30] |
| AdhereTech | Wireless pill bottle cap that acts as a proxy to an ingestion event when removed from a pill bottle. | - Real-time adherence monitoring - Well accepted by patients | | - Cannot confirm medication ingestion - Inhibited accuracy due to “pocket dosing” and “curiosity openings” - Not compatible with complex medication regimens - Potential technical malfunctions | [15,30] |
| Abiogenix uBox | Wireless pill bottle cap that acts as a proxy to an ingestion event when removed from a pill bottle. | - Objectively monitors adherence in real-time | | - Cannot confirm medication ingestion | [15] |
| SIMpill | Electronic pill bottle containing a microchip that wirelessly transmits a message to a central server following a pill cap removal event. | - Real-time adherence monitoring - Respects patient autonomy - Least stigmatizing adherence monitoring method compared to other technologies | | - Inhibited accuracy due to “pocket dosing” and “curiosity openings” | [16] |
| eCap | An electronic pill bottle that records date-and-time stamps of opening events. Has twelve optional daily audiovisual medication reminders that align with patients’ dosing schedule. Patient adherence data must be downloaded from the device via a scanner. | - Objectively monitors adherence - Provides patient adherence patterns - Wireless technology - Well accepted by patients | | - No remote access to patient adherence data - Expensive - Not suitable for complex medication regimens - Cannot confirm medication ingestion - Time burden to synthesize adherence data results and patterns - Potential technical malfunctions - Potential patient privacy concerns | [1] |
| Clever Cap LITE with the Wise App | Electronic pill bottle that records opening events. Transmits patient identifier and date-and-time stamp of opening event via existing cellular networks in real-time. Wise app provides medication audiovisual reminders. | - Real-time adherence monitoring - Discrete design that protects patient privacy - Patient medication audiovisual reminders | | - Small medication storage capacity - Device size is inconvenient for carrying/traveling - Cannot confirm medication ingestion - Audiovisual reminders may annoy patient over time | [32] |
| Elucid Pill Connect System | A pill bottle device that is equipped with an automated dispense and reminders system connected to a mobile app. Within a predefined time-frame, only the correct dose of medication is released and the moment of dispense time and internal bottle temperature is recorded when patients interact with the medication reminder via the mobile app. Patient adherence data is sent in real-time to a platform accessible to HCPs/researchers. | - Real-time adherence monitoring - Patient adherence data accessible to HCPs/researchers in real-time - Internal temperature data is useful for temperature sensitive medications - App provides medication reminders and dosing schedule information - Dispense mechanism can only be opened when a dose is due - Accepted by patients | | - Device size is inconvenient for carrying/traveling - Potential technical difficulties - More difficult to use than standard drug packaging methods | [31] |
| ***Electronic Pill Boxes and Bags*** | | | | | |
| Wisepill | A wireless pill box that communicates patient adherence behavior in real-time by transmitting the patient identifier and a date-and-time stamp via cellular networks or general packet radio service (GPRS) when opened. Can store approximately 30 large or 60 small pills in two inner compartments. | - Real-time adherence monitoring - Patient adherence data accessible to HCPs/researchers in real-time - Provides patient adherence patterns - Easy to use - Wireless technology - Feasibility well established - Respects patient autonomy - Less expensive than DOT - May improve patient medication adherence behaviors | | - Cannot confirm medication ingestion - Inhibited accuracy due to “pocket dosing” and “curiosity openings” - Expensive - Limited pill storage capacity - Occurrence of data transmission lapses reported - Potential technical malfunctions - May be challenging to use with complex medication regimens - Time burden to synthesize adherence data results and patterns - Obtrusive | [1,9,10,15,16,19,27,28,38,41,47-49,53] |
| Wisepill with SMS reminders | Electronic pill box that records a date-and-time stamp for every opening event. Patient adherence data is transmitted to a secure web server via mobile signal. Device can hold up to 60 small pills and is rechargeable. SMS reminders are sent during predetermined times or in events of nonadherence. | - Real-time adherence monitoring - Small, portable size - Patient adherence data accessible to HCPs/researchers - Customizable SMS medication reminders - Easy to use - Long battery life of 6 months - May improve patient adherence behaviors | | - Cannot confirm medication ingestion - Inhibited accuracy due to “pocket dosing” and “curiosity openings” - Potential technical malfunctions - Patient privacy concerns - Does not display battery level - Long charging period when using solar charger | [39] |
| evriMED | A digital pill box containing an electronic monitor that records opening events with date-and-time stamps. Patient adherence data is sent to a central server in near-real-time. Device provides daily audiovisual medication reminders using 3 LED lights and an electronic module. | - Real-time adherence monitoring - Inexpensive - Patient adherence data accessible to HCPs/researchers in near-real-time - Long battery life of 12 months - Can store 12,000 opening events - Can store blister packs of medication - Medication reminders | | - Cannot confirm medication ingestion - Inhibited accuracy due to “pocket dosing” and “curiosity events” - Patient privacy concerns due to large size, white design, and audiovisual reminders - Not transportable - Potential technical malfunctions | [17,18,40,51,52,93] |
| CuePBox | An electronic pill box system composed of three main components: an information management system for patients (IMSP), virtual pill box and physical pill box. The physical pill box is composed of several containers, with each container connected to a switch that can record when the patient has opened the container. Patient adherence data is communicated to the IMSP through the GSM network. Provides audiovisual medication reminders | - Real-time adherence monitoring - Patient adherence data is accessible to HCPs/researchers in real-time - Audiovisual medication taking reminders - Alerts HCPs/researchers in events of patient nonadherence via SMS | | - Efficacy not established - Evaluation of device is still on-going | [42] |
| Med-eMonitor | An electronic pill organizer that contains five different compartment drawers that can store a month’s worth of up-to five different medications. When a drawer is opened, the device asks the patient if medication was taken, and if not, the reason why. Patient adherence data is stored in device memory and downloaded to a secure website during pre-set windows through a modem cradle. Device can warn patients if they are taking wrong medication. Patients can record adverse effects/side-effects of medication on the device. HCPs/researchers are informed by the device if a patient is non-adherent or is experiencing side-effects. | - Real-time adherence monitoring - Provides patient adherence patterns - Alerts HCPs/researchers in events of patient nonadherence - Provides medication reminders - Well accepted by patients - Easy to use - Wireless technology - May improve patient adherence behaviors (average adherence rate of 91% vs 72% of usual care) | | - Potentially costly - Time burden to synthesize adherence data results and patterns - Cannot confirm medication ingestion - Potential patient privacy concerns - May be challenging with complex medication regimens | [1,102] |
| MedTracker | A smart pill box that detects the lid opening of a 7-day pill reminder pill box using plungers that are embedded in each compartment. Date-and-time stamps of opening events are transferred wirelessly via Bluetooth connection to a nearby computer. | - Real-time adherence monitoring | | - Cannot confirm medication ingestion - Inhibited accuracy due to “pocket dosing” and “curiosity events” - Limited medication storage capacity | [45] |
| SmartDrawer | An RFID-based system that uses an electronic drawer filled with RFID tagged pill boxes. Device can record the type and time of pill box removal events. A removal event serves as a proxy for medication taking when the RFID pill bottle is out of range of the RFID reader. Device interface issues medication reminders and medication dose information. | - Objective monitoring of adherence - Provides medication reminders - Alerts patient if wrong medication was removed from device | | - Cannot confirm medication ingestion - Inhibited accuracy due to “pocket dosing” and “curiosity events” | [45] |
| MedMinder | A fully programmable electronic pill box with multiple compartments. Records date-and-time stamps for each compartment opening event. Patient adherence data is transmitted wirelessly via cellular technology to a central server. Device is capable of delivering audiovisual medication reminders | - Real-time adherence monitoring - Well accepted by patients - Patient adherence data accessible to HCPs/researchers in real-time - Provides medication reminders - May improve patient medication adherence behaviors | | - Cannot confirm medication ingestion - Inhibited accuracy due to “pocket dosing” and “curiosity events” - Potential technical malfunctions - Expensive - May not be well accepted by patients with lower comfortability with technology - May not be compatible with complex medication regimens | [30,44,100] |
| MedSignals | A pill box device that consists of four plastic containers. Each lid opening event is recorded with a date-and-time stamp. Patient adherence data is uploaded to a web-based server via Bluetooth on mobile phones | - Real-time adherence monitoring - Patient adherence data accessible to HCPs/researchers in real-time - Provides patient adherence patterns - Wireless technology - Well accepted by patients - Provides medication reminders - Easy to use | | - Cannot confirm medication ingestion - Inhibited accuracy due to “pocket dosing” and “curiosity events” - Expensive - Time burden to synthesize adherence data results and patterns - Patient privacy concerns - Potential technical malfunctions - May not be compatible with complex medication regimens | [1,30] |
| Jeyun Medical Co. Smart Pill Box | A smart pill box that records whether a dose is taken as well as the appropriate medication dosage and remaining pill counts. Records date-and-time of medication removal from device. A home monitoring system accumulates patient adherence data and sends it to an electronic Case Report Form (eCRF) system that is accessible by HCPs/researchers. | - Real-time adherence monitoring - Patient adherence data accessible to HCPs/researchers in real-time - Provides patient adherence patterns - Scheduled medication reminders/alerts - May improve patient adherence behaviors | | - Cannot confirm medication ingestions - Inhibited accuracy due to “pocket dosing” and “curiosity events” | [46] |
| Indigenously designed electronic medication monitor (EMM) | System is composed of a plastic box and an electronic module. Opening events are recorded as a proxy for patient adherence. The device can be programmed with medication reminders | - Objective monitor of medication adherence - Long battery life of 2 months - Daily medication reminders - Low cost | | - Patient adherence data must be uploaded manually onto computer during clinic visits - Potential technical malfunctions | [50] |
| Sensemedic Smart | An electronic pill dispenser combined with a real-time web portal. Opening events are recorded with date-and-time stamps and sent through a central server. Provides medication reminders. | - Real-time adherence monitoring - Provides medication reminders in events of nonadherence - Patient adherence data is accessible to HCPs/researchers via online portal - Provide patient adherence patterns in visual formats - Well accepted by patients | | - Cannot confirm medication ingestion - Inhibited accuracy due to “pocket dosing” and “curiosity events” - May not be compatible with complex medication regimens - Expensive - Potential technical malfunctions | [30] |
| Tel-Me-Box | Electronic pill box that records opening events as a proxy for medication intake. | - Real-time adherence monitoring - Low cost | | - Efficacy not established - Still under development - Cannot confirm medication ingestion - Inhibited accuracy due to “pocket dosing” and “curiosity events” - May not be compatible with complex medication regimens - Potential technical malfunctions | [30] |
| Online compliance monitoring system | Wireless module mounted on Weekly Dose Pack device. The calendar type pill organizer detects removal of medicine using a circuit board and conductive ink. Breaks in the circuit indicate patient removal of medication. Device opening notifies a personal server using wireless communication. | - Real-time adherence monitoring - Patient adherence data is accessible to HCPs/researchers in real-time visual formats - Reliable data transmission - Long battery life - Circuit board is removable and reusable | | - Cannot confirm medication ingestion - Barriers may affect wireless communication distance | [96] |
| Bracelet- and Self- Directed Observational Therapy (BSDOT) | A polymer composite bracelet that stores pills in 14 compartments. An opening event of a compartment is recorded with a date-and-time stamp. Patient adherence data and other physiological data (heart rate, blood pressure) is transmitted to a mobile app. Photograph validation of medication administration is required. | - Potential real-time adherence monitoring if patient chooses to send adherence data to HCPs/researchers - Damage resistant - Provides medication reminders | | - Expensive - Efficacy not well established | [89] |
| Wisebag | A soft bag with a built-in electronic unit that records each opening event with a date-and time stamp. Transmits patient adherence data via GPRS to a central server. | - Real-time adherence monitoring - High acceptability - Long battery life of 3 months | | - Cannot confirm medication ingestion - Inhibited accuracy due to “pocket dosing” and “curiosity events” - May not be compatible for complex medication regimens - Cost - Potential technical malfunctions | [27,30,41,98] |
| GlowPack | Resealable pouch that records opening events as a proxy for medication adherence. Works similar to GlowCap | - Currently in testing | | - Currently in testing | [1] |
| ***Blister Pack Technologies*** | | | | | |
| Helping Hand (HH) | Slightly arched blister card holder that records medication removal from blister pack. Possesses programmable acoustic medication reminders and nonadherence feedback through an LED light indicator system on the device. Can generate patient adherence data print-outs to see adherence patterns. | - Objective adherence monitoring - Medication intake patterns are downloadable by HCPs/researchers - Easy to use - Suitable for blister packs - Perfect functioning observed in 70-87% of HH devices - May improve patient adherence behaviors | - Cannot confirm medication ingestion - May not be compatible with complex medication regimens - Patient privacy concerns as the pills in the device are visible - Difficulty inserting the blister cards into device observed - Potential technical malfunctions - Quiet reminders - Large size | | [4,36] |
| Med-ic | An electronic radiofrequency enabled self-adhesive label that affixes to standard blister packs. A microchip and fine wire register the date and time each pill is removed from the pack. Patient adherence data is transmitted wirelessly to an internet server. | - Real-time adherence monitoring - Objective adherence monitoring - Provides patient adherence patterns - Wireless technology - Easy to assemble - Software is easy to use - User friendly | - Cannot confirm medication ingestion - Patient adherence data can be lost if blister pack is destroyed or damaged - Potential technical malfunctions that can cause false event recordings - Time burden to synthesize adherence data results and patterns - Cost | | [1,60] |
| SmartBlisters | A radiofrequency enabled self-adhesive label that affixes to standard blister packs. Uses conductive tracks on the label to detect the removal of medication and records a date-and-time stamp. Patient adherence data can be transferred to an online database via near field communication. | - Real-time adherence monitoring - Provides patient adherence patterns - Wireless technology - Feasible within pharmacy settings - Ability to monitor each individual pill - Easy to use | - Cannot confirm medication ingestion - Time burden to synthesize adherence data results and patterns - Cost - Potential technical malfunctions that can cause false event recordings - Needs improvements to device’s robustness | | [1,59] |
| Intelligent Drug Administration System (IDAS II) | Electronic devices that fit specific blister packs and records date-and-time stamps of drug removal events from the blister pack. | - Real-time adherence monitoring - Provides patient patterns of adherence - Wireless technology - Easy to use | - Cannot confirm medication ingestion - Time burden to synthesize adherence data results and patterns - Cost - Potential technical malfunctions | | [1] |
| Cerepak | Electronic blister pack available in multiple sizes. Contains a hidden microprocessor and conductive ink tracks to record the date-and-time of pill removal events from the blister pack. | - Real-time adherence monitoring - Provides patient patterns of adherence - Wireless technology - Easy to use | - Cannot confirm medication ingestion - Time burden to synthesize adherence data results and patterns - Cost - Potential technical malfunctions | | [1] |
| Blister pack organizer with conductive ink | Conductive pattern on a blister pack pill organizer surface that detects when pills are removed due to a disconnection of the conductive track. Records the location and time opening of each pill removal event | - Real-time adherence monitoring - Compatible with multiple drugs with various administration times - Provides medication reminders - Alerts HCPs/researchers in events nonadherence events - May improve patient adherence behaviors | - Cannot confirm medication ingestion - Indirect method of adherence monitoring | | [55] |
| Near Field Communication (NFC) enabled smart medication blister | A blister pack with a conductive track that can detect pill removal events and records date-and-time stamps. Patient adherence data is collected by mobile phones using NFC. Data is then transferred to a remote telemonitoring service. | - Real-time adherence monitoring - Patient adherence data accessible to HCPs/researchers in real-time - Feasible with elderly populations | - Cannot confirm medication ingestion - Potential technical malfunctions in data transmission - Patient must own a mobile phone | | [58] |
| DigiSpenser | A simple user interface and a slot to insert a single blister pack. Programmable red/green lights indicate which compartment to open. Medication removal events are recorded as a proxy to medication adherence. | - Real-time adherence monitoring - Provides visual medication reminders - Patient adherence data is accessible to HCPs/researchers in real-time - Portable - Battery operated | - Cannot confirm medication ingestion - Device is difficult to seal and clean - Heavy and bulky - Interface needs simplification - Cost | | [57] |
| Polymedication Electronic Monitoring System (POEMS) | Self-adhesive polymer film with loops of conductive wires applied to a standard blister pack. Removal of medication breaks the conductive wire and records a date-and-time stamp. Patient adherence data is transferred via wireless communication to an online database. | - Real-time adherence monitoring - Objective monitoring of adherence - Patient adherence data is accessible to HCPs/researchers in real-time - Unobtrusive - Non-invasive - Easy to use | - Cannot confirm medication ingestion - Limited medication storage capacity - Potential patient privacy concerns | | [6,9] |
| eDosette | Internet enabled device that takes pictures of patient’s blister pack every hour. Creates medication administration records based off of medication data and photos. | - Remote adherence monitoring - Capture accuracy of 95% - Easy to use - May improve patient adherence behaviors | - Cannot confirm medication ingestion - Requires an internet connection - Cannot accommodate all blister packs and dosettes currently available - Large and not portable - Requires specific lighting conditions during picture capture period | | [54] |
| ***Ingestible Sensors*** | | | | | |
| MyTMed | Digital pill that consists of a standard gelatin pill capsule with a unique radiofrequency emitting tag. Patient medication is ingested with the sensor. Contact with gastric fluids activates the radiofrequency tag to send a signal to a hip-mounted server as an indicator of patient adherence. Patient adherence data is uploaded to an external web server. | - Real-time adherence monitoring - Direct monitoring of adherence - Patient adherence data is accessible by HCPs/researchers - Provides patient adherence patterns - Can distinguish between multiple ingestion events - Functional in low resource settings - Uses a HIPAA compliant cloud web server | | - Potential risk of radiofrequency tag retention in the body - Feasibility in real-world settings not established | [63] |
| Ingestible Sensor System (ISS) | Composed of an ingestible microsensor that becomes activated after ingestion and an adhesive personal monitor that detects medication ingestion events. Gastric fluid activates the sensor to release a signal detectable by the external monitor. Patient adherence data is uploaded to a central server via a paired mobile app. | - Real-time adherence monitoring - Direct monitoring of adherence - Patient adherence data accessible by HCPs/researchers - FDA approved - Ability to detect two ingestion events at the same time - Consistent performance observed over a 12-week period | | - Adhesive external monitor may cause skin irritation - Patient privacy concerns - May affect patient autonomy - Patient requires access to a smartphone | [52,62,64,69] |
| Proteus | Consists of a 1.0 mm by 1.0 mm ingestible sensor co-encapsulated with patient medication and an on-body wearable sensor. The ingestible sensors are activated by contact with gastric fluid and transmit unique identifying signals to the external sensor. Date-and-time of ingestion events are recorded and wirelessly transferred to the patient's mobile phone and then to a central server via cellular networks. | - Real-time adherence monitoring - Direct monitor of adherence - Patient adherence data accessible to HCPs/researchers in real-time - Utilize different radio frequencies that other wireless commercial devices - Provides patient adherence patterns - FDA approved - May improve patient adherence behaviors - Ingestion event detection accuracy of 95% | | - Adhesive external monitor may cause skin irritation - Potential risk of radiofrequency tag retention in the body - Potential risk of medication dissolution - Invasive - Patient privacy concerns - External monitor is uncomfortable and potentially stigmatizing - Large pill size due to gelatin capsule - Requires modification of medication | [9,13,15,16,49,65,66] |
| Abilify | Medication co-encapsulated with a digital sensor that communicates with an externally worn patch after ingestion. Patient adherence data is transmitted to the patient's mobile phone. | - Real-time adherence monitoring - Direct monitor of adherence - Patient adherence data accessible to HCPs/researchers - Moderate acceptability | | - Adhesive external monitor may cause skin irritation - Disregards patient autonomy - Patient privacy concerns - Cost is higher compared to standard medication | [12,21] |
| Digital Medicine System (DMS) | Consists of an ingestible sensor embedded within medication tablets, a wearable monitor, and secure mobile and cloud-based apps. Contact of the ingestible sensor with gastric fluids transmits a signal to the wearable monitor. Patient adherence data is transmitted from the external monitor to a mobile app and then to a central server. | - Real-time adherence monitoring - Direct monitor of adherence - Ingestion event detection accuracy of 97% - Moderate acceptability among patients | | - Potential technical malfunctions - Costs are unknown - Requires access to a smartphone | [3,30,75] |
| Helius | An ingestible sensor that is attached to medication pills. The sensor is activated upon interaction with stomach fluid and sends a signal to a monitor worn on the patient’s torso. Patient adherence data is uploaded to a secure server via Bluetooth. External monitor can be worn for 7 days. | - Real-time adherence monitoring - Direct monitor of adherence - Provides patient adherence patterns - Wireless technology - More convenient than DOT - Less expensive than DOT | | - Potentially costly - Patient privacy concerns - Risk of skin irritation from external monitor - Efficacy not established - Time burden to synthesize adherence results and patterns - Potential technical malfunctions | [1] |
| ID-Cap | A wearable hub that attaches to the patient’s wrist, arm, hip or abdomen that detects when a medication co-encapsulated with an ingestible sensor has been ingested. Patient adherence data is transmitted wirelessly to a central server via cellular networks. | - Real-time adherence monitoring - Direct monitor of adherence - Ingestion event detection accuracy of 97.75% - Utilizes different radio frequencies that other commercial wireless devices | | - Risk of skin irritation from adhesive external monitor - Patient privacy concerns - Limited external monitor battery life - Feasibility not established in real-world settings - Potential risk of radiofrequency tag retention in the body - Risk of medication dissolution - Potential technical malfunctions | [15,16,67] |
| Fluorophore sensor | An electronic wristwatch that detects medication containing fluorophore in the bloodstream | - Detects actual ingestion event and dosage of medication - Direct monitor of adherence | | - Long-term side-effects of fluorophore not well understood - Requires modification of medication - Invasive | [13] |
| Unidentified ingestible sensor technologies | Microsensors incorporated into medication tablets are activated upon contact with gastric fluids, sending a unique and private signal to an external sensor worn on the patient. Patient adherence data is transmitted to a central server via mobile networks or mobile app. | - Real-time adherence monitoring - Direct monitor of adherence - High accuracy - Patient adherence data is accessible to HCPs/researchers - May improve patient adherence behaviors | | - Usability and acceptability in real-world settings not established - Poor battery life - Potential technical malfunctions | [37,61,68] |
| **Electronic Medication Management Systems** | | | | | |
| Mobile Treatment Monitoring System (MTMS) | A centralized system composed of an electronic health record system, patient notification system and medication dispenser. Patient presses a button on the medication dispenser to release medication. The medication dispenser creates patient adherence feedback that is viewable by HCPs/researchers. | - Real-time adherence monitoring - Patient adherence data is viewable by HCPS/researchers - Low cost - Can be used as a communication tool between patients and HCPs/researchers - Small size | | - Cannot confirm medication ingestion - Efficacy not established - Under development | [104] |
| RFID-based Medication Adherence Intelligence System (RMAIS) | Composed of a RFID reader, scale, microcontroller, liquid crystal display (LCD) panel and motorized rotation platform. Patient’s pill bottles are labeled with an RFID tag that stores the medication’s information, such as medication name and the appropriate dose. Generates audio medication reminders at scheduled times. The scale underneath the rotation platform weighs the pill bottle and the medication information is displayed via the RFID reader After the patient has removed medication from the pill bottle, the scale re-measures the weight of the bottle and uses the difference in weight to determine the number of doses removed. HCPs are alerted during events of nonadherence. | - Remote adherence monitoring - Can determine the amount of medication removed by patient - Guides patient to take correct medication - High accuracy - Low cost - Practical - Provides medication reminders | | - Cannot confirm medication ingestion - Potential technical malfunctions - May be difficult to use among elderly populations | [45,71] |
| A proposed medication adherence monitoring system | Consists of a dosage unit and PC Kinect visual sensors, speakers, Reed-switch sensors, micro-actuators and LEDs to assess and supervise patient medication activity. Dosage unit unlocks compartments filled with medication based on patient dosing schedule. Uses video sensors to confirm medication ingestion. Sends text messages concerning patient adherence to caregiver. | - Direct monitor of adherence - Remote adherence monitoring - Guides patient to take medication correctly - Prevents access to incorrect medication - Displays patient adherence history | | - Requires caregiver to unlock dosage unit and fill medication into allocated compartments | [94] |
| Smartphone-based Medication Self-management System (SMSS) | A specialized smartphone programmed to display patient medication history, current prescriptions, medication-taking records, calendar, adherence rate information, numbers of remaining medications, and health records. Smartphone system is paired with a wireless electronic pillbox that records opening events as a proxy for medication ingestion. | - Real-time medication adherence - Adherence data is accessible to HCPs/researchers - Provides medication reminders after events of nonadherence - Uses two pillboxes: one for at home and a smaller, portable one - Cost | | - Cannot confirm medication ingestion - Medication reminders were found to be ineffective | [95] |
| MedLink | A Smartphone app that provides patient education, monitors patient adherence and provides medication reminders in events of nonadherence. The app also can also monitor medication side-effects and facilitates HCP-patient communication. The app pairs with the Wisepill device for adherence monitoring. | - Real-time adherence monitoring - Patient adherence data accessible to HCPs/researchers - Provides medication reminders in event of nonadherence - Well accepted by patients | | - Cannot confirm medication ingestion | [103] |
| Ambient Assisted Living (ALL) system | A smart environment consisting of multiple sensors to monitor patient activities to infer patient medication adherence. The system uses a smart pill box device and uses opening events as a proxy to medication ingestion. | - Adherence detecting accuracy of 80% - Recognizes nonadherence behavior related to the time of medication administration - Sends patient reminders encouraging correct medication adherence behavior | | - Patient privacy concerns - Cannot confirm medication ingestion | [92] |
| MySafeRx | A mobile app platform connected with the MedicaSafe 3000 pill dispenser. The dispenser sends via unique medication release codes via the app to patients. Programmed alerts and confidential communication with Mobile Recovery Coaches is provided by the app. | - Real-time medication adherence - Patient adherence is accessible by HCPs/researchers - Acceptable among patients - MRCs directly observe medication ingestion via videoconference | | - Potential technical malfunctions - Patients may fake ingestion of medication | [99] |
| ReX | A medication management system that monitors the medication from its packaging in the pharmacy up until its administration into the patient’s mouth. System consists of a reusable drug dispensing unit, a disposable cassette, a mobile app, and a Dose-E analytics cloud system. The mouthpiece on the dispensing unit contains an anti-choke mechanism to ensure that the medication falls directly onto the patient’s tongue. | - Real-time adherence monitoring - Medication is locked in device and can only be released at the right time and in the right dose - Provides medication reminders in events of nonadherence - Easy to use - May improve patient adherence behaviors | | - Cannot confirm medication ingestion - May be difficult to use for older populations | [72] |
| ICT-based centralized monitoring system | Uses a smart pill box and sends text message alarms regarding medication missed doses, misuses, or overdoses. The electronic pill box monitors patient medication adherence. | - Real-time adherence monitoring - Objective monitoring of adherence - Safe and accurate - Well accepted by patients | | - Cannot confirm medication ingestion - Potential technical malfunctions - Data transmission difficulties in areas with inconsistent Internet connection | [101] |
| Medication Behavior Monitoring System (MBMS) | A device paired with motion sensors that includes an eight-compartment pill box and a video-camera system. Medication is dispensed following correct movements detected by the video camera and motion sensor systems. Medication ingestion is identified through the use of a scale system that records an ingestion event if the scale reads zero after medication has been dispensed. | - Prevents underdosing or overdosing of medication - High accuracy - Adherence data accessible to HCPs weekly - Medication reminder alarms | | - Cannot confirm medication ingestion - Noted minor difficulties dispensing flat pills | [73] |
| Evondos E300 | A robotic device that integrates medication distribution, supports individualized dosing schedule, provides medication reminders and acts as a communication tool with HCPs. The medication reminder messages are audiovisual and indicate when the patient should press the medication dispensing button. Doses of medication are locked if not retrieved within a predetermined time frame. HCPs receive alerts in event of untaken doses. | - Real-time adherence monitoring - Monitors medication dose by dose - Alerts HCPs in events of nonadherence - Patient can only access medication within predetermined time frames which prevents taking medication at the wrong time - Well accepted by patients | | - Cannot confirm medication ingestion - Requires medication to be packaged in individual dose sachets in the pharmacy - Potential technical malfunctions | [97] |
| ***Video-based Adherence Monitoring Technology*** | | | | | |
| Mobile Direct Observation of Therapy (MDOT) | A mobile device is used to self-record videos of patients’ administering their medication. Videos are sent electronically to be reviewed by HCPs/researchers | - Real-time adherence monitoring - Cost effective compared to DOT - Reduced burden on patients and HCPs compared to DOT - Flexible and convenient - May improve patient adherence behaviors | | - Cannot always confirm ingestion of medication - Poor video quality limits the ability to assess adherence - Potential technical difficulties during video uploading process - Patient privacy concerns - Patient may forget to take a video while administering medication - Requires adequate mobile telephone network coverage | [77] |
| Unique camera system | The system detects interactions between patient hands and medication pill bottles, and interactions between the hands and the patient’s face to track and detect medication intake events. | - Medication intake event detection accuracy of 75% - Patient’s face and hands are traceable in 94% of observed frames | | - Assumes that the patient is cooperative and swallowing medication - Clothing and accessories can inhibit the systems tracking abilities - Does not track patient’s mouth - Cannot recognize patient | [74] |
| Video Directly Observed Therapy (VDOT) | Patients record videos of themselves administering medication via webcam or smartphone for remote verification by HCPs/researchers. Videos can be synchronously or asynchronously taken. Asynchronous videos are uploaded to a secure cloud server for HCPs/researchers review | - Real-time adherence monitoring - Easy to use - Less intrusive than DOT - Greater flexibility than DOT - Offers high confidence adherence data | | - May overreport patient adherence - Requires smartphone or webcam and internet access - Patient privacy concerns - Potential technical difficulties with system | [16,18,78] |
| SureAdhere | An asynchronous VDOT system that uses a smartphone or webcam | - Real-time adherence monitoring - Direct observation of ingestion events - Less expensive than DOT | | - Requires access to smartphone or webcam - Patient privacy concerns | [52] |
| AiCure | A mobile app system that uses CV algorithms to confirm pill ingestion in real-time. Patients take a video of medication administration and the technology identifies that patient and type of drug in real-time. | - Real-time adherence monitoring - Direct observation of medication ingestion | | - Patient privacy concerns - Requires access to a smartphone | [75,76] |
| iMEC | Ubiquitous sensors are attached to home appliances to predict patient adherence behavior. A medication pill bottle is also equipped with a camera to detect pill removal. | - Objective monitor of adherence | | - Cannot confirm medication ingestion - Patient privacy concerns | [45] |
| ***Motion-Sensor Technology*** | | | | | |
| Smart Medication Alert and Treatment Electronic Systems (SmartMATES) | Non-intrusive wearable sensors worn on the patient’s wrists. Within the sensors, two 3-axis accelerometers to track the left- and right-hand gestures to identify medication administration behaviors. System recognizes when patients fail to take medication and sends medication reminder alerts to patients. | - Real-time adherence monitoring - Medication reminders in event of nonadherence - Non-intrusive - Can differentiate between medication taking actions and other daily living actions | | - Only tested within a small sample size over a short period of time | [80] |
| Wireless wearable accelerometers | Wireless wearable sensors worn on patient’s wrists. Uses tri-axial accelerometers to identify patient medication administration behaviors. | - Cost effective - Non-invasive - Positive event detection rate of 84.17% | | - Actions that resemble medication administration movements may produce inaccurate medication intake readings | [13] |
| Smartwatch-based medication intake detection system | Consists of a standard smartwatch, smartwatch application and cloud-based distributed data storage and processing pipeline. Smartwatch contains a 3-axis accelerometer, gyroscope, near-field communication (NFC) and heart rate monitor. Smartwatch app collects accelerometer and gyroscope data. Uses machine learning algorithms to classify patient medication intake activity. Patient adherence data is stored in a database. | - Real-time adherence monitoring - High accuracy - Non-invasive - Low-cost | | - Potential for algorithms to misclassify patient movements | [79] |
| ***Patient Self-Report Technology*** | | | | | |
| Interactive Voice Response (IVR) | Telephone-based technology that used pre-recorded messages to collect information on patient adherence through voice and keypad inputs. Records the date, time and duration of each phone call. Uses SMS to collect adherence data through text messages. | - Real-time adherence monitoring - Patient adherence data is accessible to HCPs/researchers - Feasibility established in real-world settings - May improve patient adherence patterns | | - Subjective method of adherence monitoring - May create false estimates of medication adherence due to patient reporting bias - Requires access to a mobile phone - Cost | [38,81,84,105] |
| Telesage | An interactive voice response system. Patients answer adherence questions over a phone call using keypad inputs. | - Real-time adherence monitoring - Patient adherence data is accessible to HCPs/researchers | | - Requires access to a mobile phone - May create false estimates of medication adherence due to patient reporting bias - Expensive compared to traditional self-report | [82] |
| SIMmed | Patient places a free call or sends an SMS to a central server after ingesting medication | - Real-time adherence monitoring - Patient adherence data is accessible to HCPs/researchers - Non-stigmatizing - Respects patient autonomy | | - May create false estimates of medication adherence due to patient reporting bias | [16] |
| Medication Module™ with the LogPad® LW handheld device | Patients enter medication data via the LogPad including the date and time of medication intake events | - Easy to use - Real-time adherence monitoring | | - May create false estimates of medication adherence due to patient reporting bias | [85] |
| Smart Button system | System consists of a commercially available smart button called Stone and its companion app, Pebblebee. The smart button is programmed so that a push indicates a medication intake event. The app records date-and-time stamps of each button press and uploads them to a cloud-based server. A computer algorithm generates patient feedback via SMS based off of the patient’s dosing schedule. | - Real-time adherence monitoring - Small design - Long battery life of 1 year | | - Requires access to a smartphone - May create false estimates of medication adherence due to patient reporting bias - Currently the app must be running in the background of the mobile device for the smart button to work - May not work outside of the United States - Potential technical difficulties | [88] |
| Smart about medications (SAM) platform | An online platform that contains medication information, side-effect identification tool, medication adherence monitoring and alerts, and a communication portal with a hospital pharmacist. Adherence alerts are generated using decision algorithms and pharmacy claims data. | - Accepted by patients - Interoperability with insurance systems - Compatible with multiple medications and complex regimens - Medication adherence data is accessible to HCPs, caregivers, and patients | | - Not yet tested in real-world settings - May create false estimates of medication adherence due to patient reporting bias - Requires access to computer and internet | [86] |
| TrackYourMed | A mobile app that sends medication taking reminders. QR codes provided by HCPs are printed as stickers and can be scanned by the app to indicate medication taking events. | - Compatible with multiple medications and complex regimens - Medication adherence data is accessible to both HCPs and patients - Good patient acceptability | | - Requires access to smartphone - May create false estimates of medication adherence due to patient reporting bias - Cannot confirm medication ingestion | [87] |
| 99DOTS | A custom blister pack envelope that contains hidden random toll-free phone numbers that are only revealed when medication is removed. Patients call the revealed phone numbers to confirm medication ingestion. Each phone call is recorded as a medication taking event. | - Real-time adherence monitoring - Feasibility established in real-world settings - Less expensive than DOT - Respects patient autonomy - Patient adherence data is accessible to HCPs/researchers - Reduces burden on patients and HCPs/researchers compared to DOT | | - May create false estimates of medication adherence due to patient reporting bias - Cannot confirm medication ingestion - Patient privacy concerns - Requires access to a phone and cellular network | [16,18,19,52,56,83] |
